# Tissue Type: A Crucial Factor Influencing the Fungal Diversity and Communities in Sichuan Pork Bacon

**DOI:** 10.3389/fmicb.2021.655500

**Published:** 2021-06-25

**Authors:** Miao Zhang, Haijun Qiao, Weibing Zhang, Zhongming Zhang, Pengchen Wen, Yan Zhu

**Affiliations:** ^1^College of Food Science and Engineering, Gansu Agricultural University, Lanzhou, China; ^2^College of Science, Gansu Agricultural University, Lanzhou, China

**Keywords:** traditional pork bacon, tissue, high-throughput sequencing, physicochemical characteristics, microbial interaction

## Abstract

This study aimed to the variations of fungal diversity and community structure in different parts of traditional homemade Sichuan pork bacon. A total of seven phyla and 91 fungal genera were identified. Among them, Ascomycota and Basidiomycota were the first and second most abundant phyla in the bacon tissues. In addition, five dominant genera (*Aspergillus, Candida, Debaryomyces, Malassezia*, and *Penicillium*) were shared by all bacon tissues. The numbers of OTUs unique to individual groups were 14, 67, and 65 for the muscle tissue, the adipose tissue, and pork skin, respectively. Linear discriminant analysis showed that a total of 31 taxa significantly differed among the groups. Results of redundancy analysis indicated that fat content, protein content, aw, and pH of bacon tissue shaped the bacon fungal communities. Results of network analysis also indicated that tissue type was a crucial factor influencing the fungal interactions in different tissues. This study can lay a foundation for further isolation and identification of fungi in the product and provides a basis for further research of food health in homemade traditional pork bacon.

## Introduction

Chinese pork bacon, a famous traditional meat product, possesses a long history in southern China (Wang et al., 2019; Huang et al., 2020). A variety of traditional Chinese pork bacon styles such as Hunan, Sichuan, Guangdong, Jiangxi, and Yunnan styles have been produced and favored by local people (Yu and Sun, 2005). Among them, Sichuan-style is one of the most famous smoked styles (Guo et al., 2016; Wang et al., 2019). The traditional preparation process of Sichuan pork bacon generally consists of cutting, curing, smoking, and ripening. It is usually prepared in the winter of each year to ensure an adequate supply of meat available for next spring (Guo et al., 2016). Due to its delicious taste and flavor, Sichuan pork bacon is being favored by many people in China (Xie et al., 2008; Guo et al., 2016).

The quality of traditional meat products is often greatly influenced by environmental microorganisms (Bernardi et al., 2019). During the production of these products, the surface of the raw meat is exposed to the environment, so naturally-occurring microorganisms may colonize (Doulgeraki et al., 2012). Abundant and diverse fungal populations, including *Aspergillus*, *Mucor*, *Penicillium*, and yeast have been found in this specific food ecosystem (Mendoza et al., 2014; Magistà et al., 2017). Some of them can endow meat products with good flavor, anti-oxidative effects and protection role against detrimental microorganisms (Magistà et al., 2017; Ryu et al., 2018; Rodrigues et al., 2019). Some can also lead to undesirable effects, such as unsightly colored spots, off-flavors or toxic fungal metabolites (Ryu et al., 2018; Rodrigues et al., 2019). Because of the lack of information concerning fungi on the surface of Sichuan pork bacon, no quality standards regarding these organisms exist. Therefore, it’s necessary to investigate the fungal communities of this product.

In the production of bacon, fresh streaky pork is often used as the raw material, which includes the muscle tissue, the adipose tissue, and pork skin. Because of obvious differences in the tissue status and physicochemical properties of these different parts, they may affect the species of attached microorganisms (Ellis et al., 1983). Previous studies have shown that climate, geography, and processing technology have impacted the bacterial communities in the bacons (Yi et al., 2016). However, there have been few reports on the effect of tissue type of raw meat on the fungal community structure in traditional homemade pork bacon. In this study, our objective was to make an inventory of the diversity and interactions of the fungal communities in different tissues of homemade traditional bacon using Illumina Miseq metabarcoding.

## Materials and Methods

### Sample Collection

**FIGURE 1:**
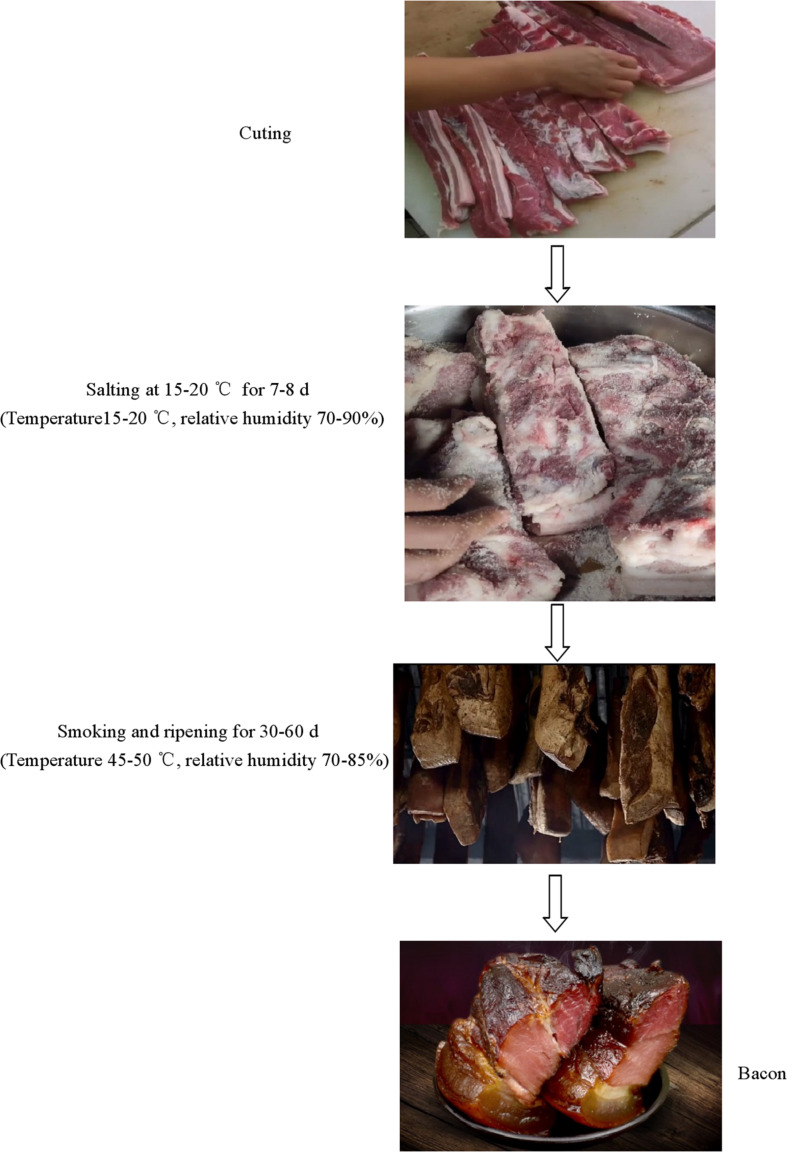
A schematic diagram of the production of homemade pork bacon.

Homemade bacon samples were collected directly from six local producers in Lacquer Tree village, Fan Kuai town, Xuanhan County and Dazhou City (Sichuan Province, China). The processing technology of the bacon is presented in Figure 1. Five bacon sticks were randomly collected from each producer. The samples were packed in sterile bags and transported to the laboratory. In sampling, bacon tissues around 2 mm from the surface were sliced using a sterilized knife. Each bacon sample was divided into three parts based on the tissue of bacon; the first part included the sample from the adipose tissue (F), the second part included the sample from the muscle tissue (M), and the third part included the sample from the pork skin (P). Then, newly collected samples were directly used for physicochemical and microbiological analysis and the samples for high-throughput sequencing were stored at –80°C.

### Physicochemical Analyses

Protein, fat, and moisture content were determined according to the Chinese Standard GB 5009.5-2016, GB 5009.6-2016, and GB 5009.3-2016 procedures, respectively (Guo et al., 2016). Salt content was measured on the basis of the Chinese Standard GB 5009.42-2016 procedures (Guo et al., 2016). a_w_ (water activity) was determined using a water activity meter (Novasina, Switzerland) while pH was measured using a digital pH meter (Sartorius, Germany).

### Microbial Enumeration

Microbial enumeration was conducted according to the method described by Li et al. (2019). Samples (25 g) were added aseptically to 225 mL of sterile Buffered Peptone Water (Merck, Darmstadt, Germany), and homogenized for 2 min within a stomacher (Interscience, Saint Nom la Bretèche, France). Decimal dilutions were prepared in Ringer’s solution (LabM, Bury, United Kingdom) for microbial enumeration. Then aliquots of 0.1 mL were spread on the following growth media for the following microbial viable counts: Aerobic plate counts on Plate Count Agar (PCA, LabM) incubated at 30°C for 3 days; Xerophilic fungi on Dichloran 18% Glycerol Agar (DG18, Hopebio), incubated at 25°C for 5 days; Yeasts and molds on Rose Bengal (RB, Lang Bridge), incubated at 25°C for 5 days.

### DNA Extraction, PCR Amplification, and Sequencing

Each sample (25 g) was homogenized with 225 mL of peptone saline solution (0.85% NaCl and 0.1% peptone in distilled water) for 30 min within a stomacher. 20 mL quantities of the solution were centrifuged for 10 min at 9,000 × *g*, and the pellet was used to extract genomic DNA with a Soil DNA Kit D5625-01 (Power Soil DNA Isolation kit, MOBIO Laboratories, Inc., United States) according to the manufacturer’s instructions.

The ITS rDNA gene targeting the ITS1-ITS2 region was amplified using the primer pairs ITS1-F (CTTGGTCATT TAGAGGAAGTAA) and ITS2 (GCTGCGTTCTTCATCGA TGC), as previously described (Luo et al., 2020). Subsequently, the library was constructed and the isolated DNA was sequenced on an Illumina Miseq PE300 Sequencing platform (Illumina, Inc., San Diego, CA, United States) at Biomarker Technologies Corporation (Beijing, China). Then, the sequencing data were uploaded into the Sequence Read Archive at NCBI under the accession number PRJNA610642.

### Processing of High-Throughput Sequencing Data

The pair-end reads from raw DNA sequences were conducted using the FLASH software (Magoc and Salzberg, 2011). Data quality control and analysis were mainly conducted using the software package of QIIME^1^ (Caporaso et al., 2010). The high-quality sequences were obtained by discarding the low-quality sequences from raw data, including sequences shorter than 150 bp, with any ambiguous bases, containing mononucleotide repeats more than 6, Phred score below 25, or chimeric sequences (by QIIME). Quality sequences were classified into operational taxonomic units (OTUs) with a cutoff of 97% identity, by the QIIME-uclust OTU-picking workflow. Taxonomies were annotated using the UNITE databases (Koljalg et al., 2013) by a QIIME-based wrapper of RDP-classfier (v.2.2). To minimize the difference in sequencing depth across samples, an averaged, rounded, and rarefied OTU table was generated by averaging 100 evenly re-sampled OTU subsets under the 90% of the minimum sequencing depth. Alpha diversity indices of Chao1, Shannon, and Good’s coverage for the tissues were calculated using QIIME(V 1.9.1). Non-metric Multidimensional Scaling (NMDS) was conducted to analyze the differences between the tissues using R software (version 2.15.3). The Adonis permutational multivariate analysis (Adonis/PERMANOVA) were used to test the statistical difference of fungal structure among the tissues (Lozupone et al., 2007). LEfSe (Linear discriminant analysis Effect Size) was conducted to identify differentially abundant taxa between different tissues (LDA score ≥3.0 and *p* ≤ 0.05) (Segata et al., 2011). The SparCC algorithm was used to analyze the correlation of fungal genera in the bacon tissues, including positive correlation and negative correlation (Friedman and Alm, 2012). Statistical analysis was also performed on the screening of the correlation score >0.6 with a significance level less than 0.05. Networks analysis was conducted using the biocloud tools, as previously described (Luo et al., 2020). Redundancy analysis (RDA) was also implemented using the biocloud tools.

### Statistical Analysis

Differences of physicochemical properties, microbial counts and fungal diversity between the tissues were evaluated using the Duncan’s test. *P*-values below 0.05 were considered considered as being statistically significant.

## Results

### Physicochemical Analyses and Microbial Enumeration

The pH of bacon ranged from 5.68 to 5.78; however, no significant difference (***P*** > 0.05) was found between the tissues (Table 1). Protein content, salt content, and a_w_ of the adipose tissue were significantly lower than those of the other tissues (***P*** < 0.05). Fat content of the tissues ranged within 24.57–84.06 g per 100 g, with the lowest and highest values from the muscle and adipose tissues, respectively. Moisture content of the adipose tissue was lowest (*P* < 0.05). The aerobic plate count on the PCA was lowest (5.27 ± 0.09 log10 CFU per g) in the muscle tissue (Table 2). There were significantly fewer xerophilic fungi for the muscle tissue on the DG18 (1.95 ± 0.08 log10 CFU per g) than that for the two other tissues (***P*** < 0.05). Additionally, molds and yeasts on the RB was lowest (1.43 ± 0.04 log10 CFU per g) in the muscle tissue (Table 2). No significant difference (***P*** > 0.05) was found for aerobic plate count on the PCA, molds and yeasts on the RB, and xerophilic fungi on the DG18 between the adipose tissue and pork skin.

**TABLE 1:** Physiochemical characterization of bacon tissues.

Group	Protein content (g per 100 g)	Fat content (g per 100 g)	Moisture content (g per 100 g)	Salt content (g per 100 g)	a_w_	pH
M_mean_	53.49 ± 2.48^a^	5.54 ± 0.23^*c*^	34.59 ± 2.56^ab^	5.36 ± 0.19^a^	0.84 ± 0.02^a^	5.78 ± 0.11^a^
F_mean_	5.25 ± 0.13^*c*^	84.06 ± 1.80^a^	8.22 ± 0.16^b^	2.31 ± 0.14^b^	0.77 ± 0.02^b^	5.68 ± 0.19^a^
P_mean_	29.29 ± 1.42^b^	24.57 ± 1.48^b^	36.49 ± 1.52^a^	5.41 ± 0.26^a^	0.84 ± 0.04^a^	5.72 ± 0.21^a^

*Letters indicate Duncan’s pairwise differences among different tissues (*p* < 0.05).*

*M, sample from the muscle tissue of bacon; F, sample from the adipose tissue of bacon; P, sample from pork skin of bacon.*

**TABLE 2:** Microbial enumeration of the different bacon tissues.

Group	Aerobic plate count log_10_ CFU/g	Xerophilic fungi log_10_ CFU/g	Molds and yeasts log_10_ CFU/g
M_mean_	5.27 ± 0.09^b^	1.95 ± 0.08^b^	1.43 ± 0.04^b^
F_mean_	6.58 ± 0.07^a^	3.88 ± 0.11^a^	2.85 ± 0.06^a^
P_mean_	6.77 ± 0.09^a^	3.90 ± 0.08^a^	2.91 ± 0.05^a^

*M, sample from muscle tissue of bacon; F, sample from adipose tissue of bacon; P, sample from pork skin of bacon.*

### Sequencing and Analysis of Alpha Diversity

After quality filtering and chimera removal, 153,880 high quality sequencing reads (average = 8,548 reads per sample) were obtained from 18 bacon samples, with the average read length of 211 bases for fungi. A total of 1,448 operational taxonomic units (OTUs) were generated from the high-quality sequences, with an average of 80 OTUs of individual samples (Table 3). The OTU richness was significantly lower in the muscle tissue (47 OTUs on average) than in the other two tissues (95 and 98 OTU richness for the adipose tissue and pork skin, respectively) (***P*** < 0.05). However, OTUs did not significantly differ between the adipose tissue and pork skin (***P*** > 0.05). Fungalcommunity richness and diversity were assessed using three alpha-diversity metrics (Shannon, Chao1, and Good’s coverage) (Table 3). The smallest and largest Shannon indexes were 1.51 and 3.37 for the muscle tissue and pork skin on average, respectively. The results indicated that fungal diversity was lowest in the muscle tissue. The smallest Chao1 index was 93.1 for the muscle tissue on average.

**TABLE 3:** Reads, OTUs, Good’s coverage, Chao1, and Shannon’s indices for ITS rRNA sequencing of the bacon tissues.

Group	Reads		OTUs		Good’s coverage		Chao1		Shannon	
	Mean	SD	Mean	SD	Mean	SD	Mean	SD	Mean	SD
M	35923	1403	47	3^b^	96.58%	0.1%	93.1	3.5^b^	1.51	0.34^*c*^
F	37873	1190	95	6^ab^	95.19%	0.4%	160.7	5.2^a^	2.94	0.35^b^
P	36962	1589	98	5^a^	94.98%	0.5%	153.9	6.1^ab^	3.37	0.39^a^

*Letters indicate Duncan’s pairwise differences among different tissues (*p* < 0.05). M, sample from the muscle tissue of bacon; F, sample from the adipose tissue of bacon; P, sample from pork skin of bacon.*

### Fungal Communities in the Bacon Tissues

There were six phyla detected in the tissues, two of which were dominant with relative abundances exceeding 1%. Ascomycota was the most abundant phylum in all tissues, with a relative abundance range of 75.61–95.53% (Table 4). The relative abundance of Ascomycota in the muscle tissue was notable for exceeding 95%. Basidiomycota was the second most predominant phylum in all tissues, accounting for only 3.13% of the sequences on average in the muscle tissue, but for 10.47 and 16.42% in the adipose tissue and pork skin, respectively. The relative abundances of the other four phyla (Glomeromycota, Mortierellomycota, Mucoromycota, and Rozellomycota) were below 1%.

**TABLE 4:** Percentage of the main fungal phylum and genera in tissues.

Fungi	Composition in tissues		
	F *n* = 6	M *n* = 6	P *n* = 6
**Phylum**			
Ascomycota	82.44%	95.53%	75.61%
Basidiomycota	10.47%	3.13%	16.42%
Cercozoa	0.05%	0.02%	0.17%
Glomeromycota	0.04%	0.01%	0.04%
Mucoromycota	0.01%	0.00%	0.01%
Rozellomycota	0.01%	0.00%	0.01%
Mortierellomycota	0.00%	0.01%	0.01%
**Genus**			
*Debaryomyces*	30.09%	67.67%	15.08%
*Aspergillus*	14.04%	13.37%	9.38%
*Candida*	8.29%	3.55%	16.30%
*Malassezia*	7.59%	2.15%	12.67%
*Penicillium*	8.91%	4.13%	4.89%
*Trichoderma*	6.81%	0.50%	1.62%
*Tausonia*	1.14%	0.56%	1.73%
*Fusicolla*	0.53%	0.73%	1.68%
*Acremonium*	0.92%	0.56%	1.37%

*M, sample from the muscle tissue of bacon; F, sample from the adipose tissue of bacon; P, sample from pork skin of bacon.*

There were 91 genera found in the bacon tissues and the relative abundances of the dominant fungal genera are shown in Table 4. A total of nine dominant genera were obtained in all tissues, with relative abundances >1%. In the muscle tissue, five dominant genera (*Debaryomyces*, *Aspergillus*, *Penicillium*, *Candida*, and *Malassezia*) were observed, with relative abundance in the range of 2.15–67.67%. Among them, *Debaryomyces* and *Aspergillus* were the first and second most abundant genera, representing 81.04% of the fungal population. In the adipose tissue, seven dominant genera (*Debaryomyces*, *Aspergillus*, *Penicillium*, *Candida*, *Malassezia*, *Trichoderma*, and *Tausonia*) occurred, with relative abundance of 30.09, 14.07, 8.91, 8.29, 7.59, 6.81, and 1.14%, respectively. In the pork skin, nine fungal genera (*Candida*, *Debaryomyces*, *Malassezia*, *Aspergillus*, *Penicillium*, *Tausonia*, *Fusicolla*, *Trichoderma*, and *Acremonium*) were dominant, with relative abundance of 16.30, 15.08, 12.67, 9.38, 4.89, 1.73, 1.68, 1.62, and 1.37%, respectively. Additionally, a total of 82 non-dominant genera (relative abundance <1%) were identified in the tissues.

### Comparison of Fungal Communities

**FIGURE 2:**
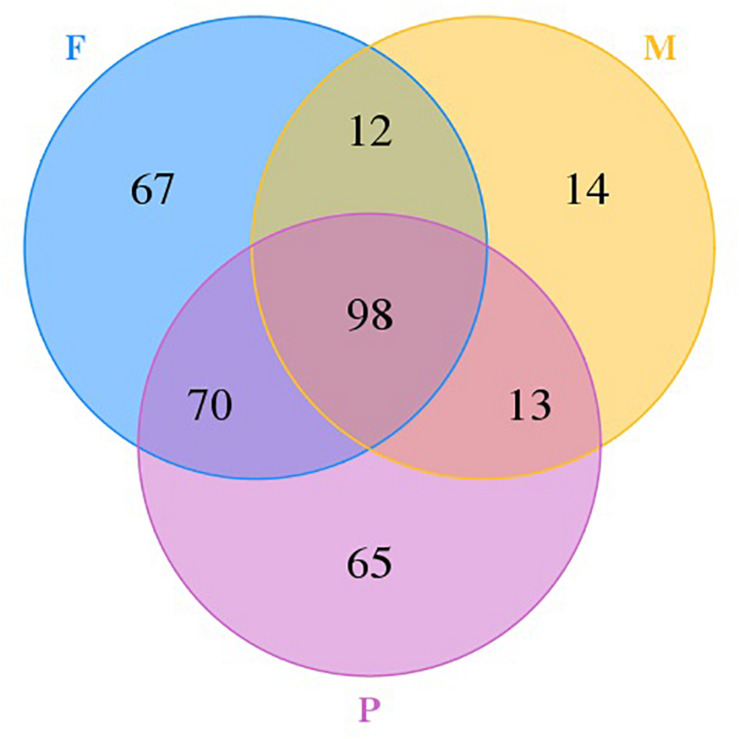
Venn diagram showing the unique and shared OTUs in different tissues.

A Venn diagram with unique and shared OTUs of the fungal communities was constructed to show the differences and similarities among the tissues (Figure 2). A total of 339 OTUs were observed, and 98 were common to all tissues. The numbers of OTUs unique to individual tissues were 14, 67, and 65 for the muscle tissue, the adipose tissue, and pork skin, respectively. The pork skin and the adipose tissue shared more OTUs (168 or 49.6% of the total) than either did with the muscle tissue.

**FIGURE 3:**
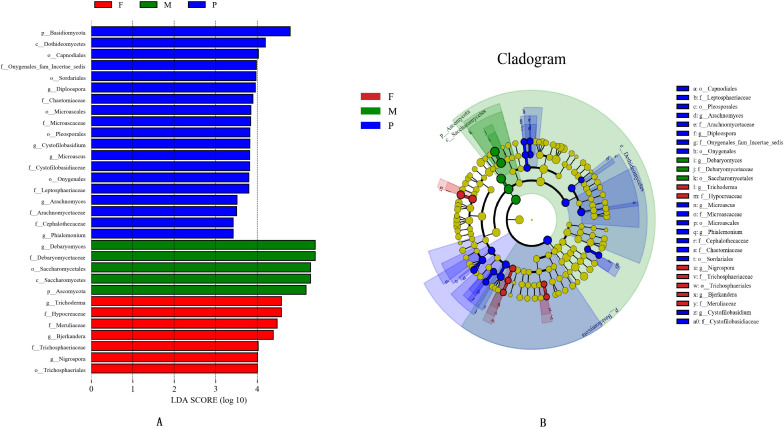
Histogram of differentially abundant features between tissues (logarithmic LDA score ≥ 4.0 and *p* ≤ 0.05) **(A)**. Cladogram indicating the phylogenetic distribution of taxon associated with the tissues of bacon (logarithmic LDA score ≥ 4.0 and *p* ≤ 0.05) **(B)**. Differences are represented in the color of the most abundant class. The diameter of each circle is proportional to the abundance of the taxon. The node size represents the difference in relative abundance.

The LEfSe analysis was applied to identify the potential distinguishable taxa among different tissues (Figures 3A,B). A cladogram showed 31 significantly different taxa between the tissues: two phyla, two classes, seven orders, eleven families, and nine genera (LDA score ≥3.0 and ***P*** ≤ 0.05; Figure 3B). In the muscle tissue, five taxa were enriched including one phylum (Ascomycota), one class (Saccharomycetes), one order (Saccharomycetales), one family (Debaryomycetaceae), and one genus (*Debaryomyces*), and all of them had an LDA value higher than 5.0 (Figure 3A). In the adipose tissue, the fungal groups enriched were one order (Trichosphaeriales), three families (Hypocreaceae, Meruliaceae, and Trichosphaeriaceae) and three genera (*Bjerkandera, Nigrospora, and Trichoderma*), both with LDA values higher than 4.0 (Figure 3A).

In the pork skin, 19 significantly different taxa were found, which included one phylum (Basidiomycota), one class (Dothideomycetes), five orders (Capnodiales, Microascales, Onygenales, Pleosporales, and Sordariales), seven families (Arachnomycetaceae, Cephalothecaceae, Chaetomiaceae, Cystofilobasidiaceae, Leptosphaeriaceae, Microascaceae, and Onygenales fam Incertae sedis), and five genera (*Arachnomyces*, *Cystofilobasidium*, *Diploospora*, *Microascus*, *Phialemonium*). Among them, 8 significantly different taxa had LDA values exceeding 4.0 (Figure 3A).

**FIGURE 4:**
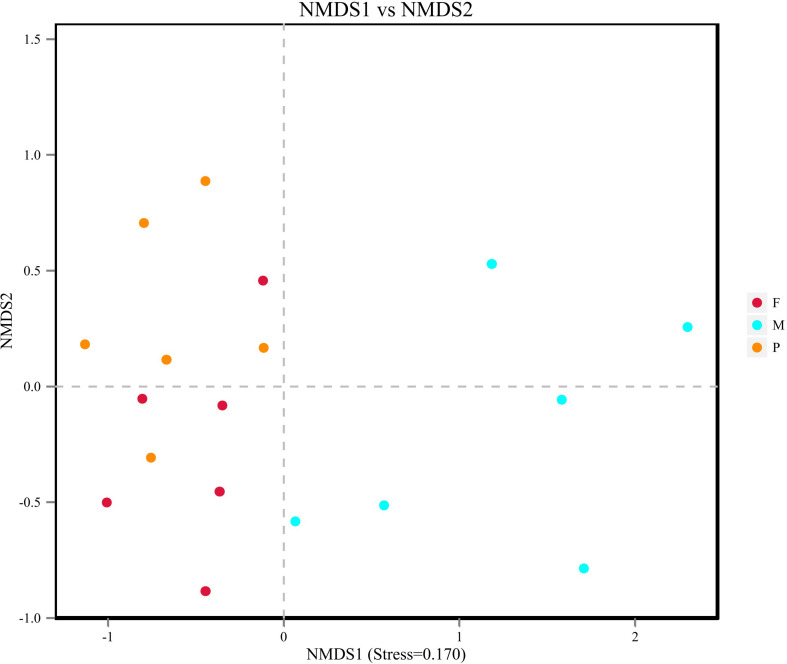
Non-metric dimensional scaling (NMDS) plot showing the association between fungal communities in bacon.

Beta diversity results of NMDS based on Jaccard distance indicated the data distribution of bacon tissues (Figure 4). Fungal communities in the adipose tissue and pork skin were more similar and located closer to each other, while the muscle tissue clearly separated. Adonis/PERMANOVA analysis performed on the tissues showed that *P* = 0.011 (*P* < 0.05), indicating that the tissue state of bacon was a crucial factor influencing the fungal composition of the different tissues.

### Interactions of Fungi in Bacon Tissues

**FIGURE 5:**
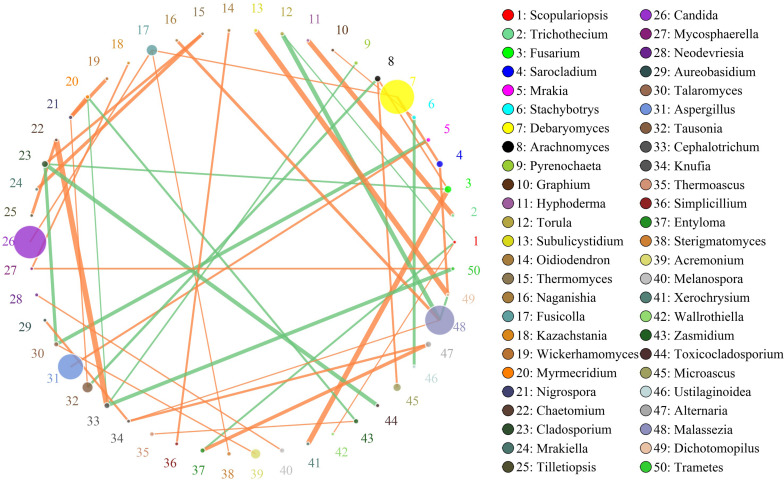
Networks of fungal interaction in the pork skin. Circle is on behalf of the genus, size of the circle represents the abundance. Lines represent the correlation between the two species, line thickness to represent the strength of the correlation (the larger the correlation coefficient, the thicker the line), the color of the line: orange represents the positive correlation, green represents the negative correlation.

In this study, the SparCC algorithm was used to calculate the relationship between genera identified in bacon and visualized as a network (v 3.7.1). The network for fungal communities in the pork skin consisted of 50 nodes and 45 edges (Figure 5). Our results suggested that the network was cooperative, and the ratio of cooperative and non-cooperative interactions was 31:14. No hub genera (≥5 edges per node) was found in the network. *Candida*, the most dominant genus in the pork skin, had a positive relationship with *Fusicolla*. All effects related to *Debaryomyces* were positive, including two other genera: *Fusicolla* and *Sarocladium*. *Malassezia* had a negative relationship with *Torula* and *Dichotomopilus*, but had a positive relationship with two other genera: *Naganishia* and *Knufia*.

**FIGURE 6:**
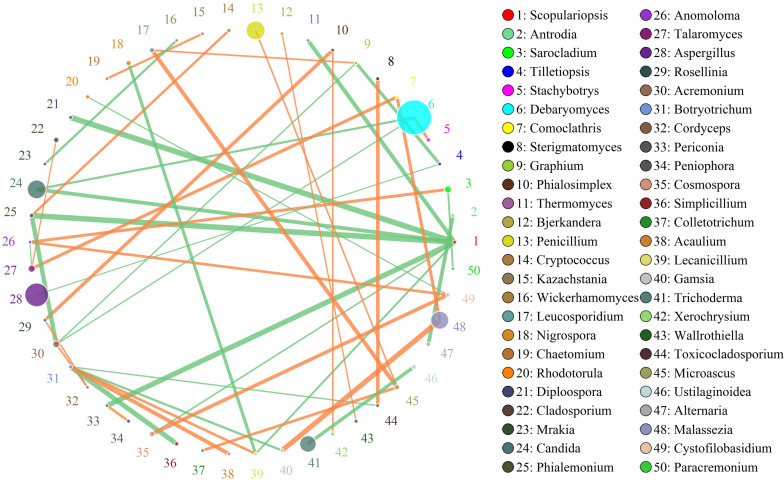
Networks of fungal interaction in the adipose tissue. Circle is on behalf of the genus, size of the circle represents the abundance. Lines represent the correlation between the two species, line thickness to represent the strength of the correlation (the larger the correlation coefficient, the thicker the line), the color of the line: orange represents the positive correlation, green represents the negative correlation.

The network for the fungal communities in the adipose tissue consisted of 50 nodes and 46 edges (Figure 6). Results suggested that the network was non-cooperative, and the ratio of cooperative and non-cooperative interactions was 21:23. *Scopulariopsis* and *Botryotrichum* were the first and second hub genera in the network. *Scopulariopsis* had a negative relationship with seven genera (*Diploospora*, *Periconia*, *Thermomyces*, *Candida*, *Lecanicillium*, *Cosmospora*, and *Phialemonium*). *Botryotrichum* had a negative relationship with *Toxicocladosporium*, *Gamsia*, and *Simplicillium*, but a positive relationship with *Acaulium* and *Lecanicillium*.

**FIGURE 7:**
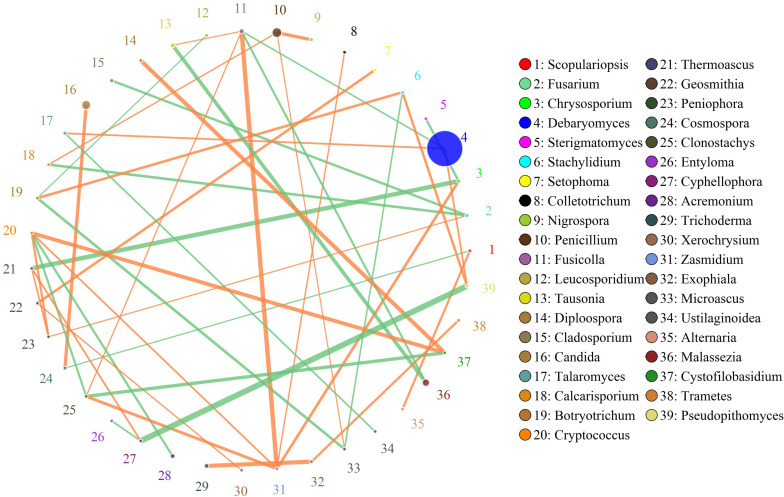
Networks of fungal interaction in the muscle tissue. Circle is on behalf of the genus, size of the circle represents the abundance. Lines represent the correlation between the two species, line thickness to represent the strength of the correlation (the larger the correlation coefficient, the thicker the line), the color of the line: orange represents the positive correlation, green represents the negative correlation.

The network for the fungal communities in the muscle tissue consisted of 39 nodes and 43 edges (Figure 7). Results suggested that the network was cooperative, and the ratio of cooperative and non-cooperative interactions was 26:17. Three hub genera (*Zasmidium*, *Fusicolla* and *Cryptococcus*) were found in the network. All effects related to *Zasmidium* were positive, including five other genera: *Clonostachys*, *Fusicolla*, *Chyrsosporium*, *Cryptococcus*, and *Colletotrichum*. *Fusicolla* had a negative relationship with *Malassezia* and *Debaryomyces*, but a positive relationship with *Zasmidium*, *Clonostachys*, and *Tausonia*. *Cryptococcus* had a negative relationship with *Clonostachys* and *Acremonium*, but a positive relationship with *Zasmidium*, *Peniophora*, and *Cystofilobasidium*.

## Discussion

### Fungal Communities in Sichuan Pork Bacon Tissues

Pork bacon, a traditional meat product, has long been popular in the south of China (Yi et al., 2016). During the production of traditional pork bacon, raw meat is processed under exposed conditions; hence, a variety of microorganisms may be involved in this specific food ecosystem. In the present study, a total of 6 fungal phyla were detected in the bacon tissues. Among them, Ascomycota and Basidiomycota were the first and second most abundant phyla in the bacon tissues. These phyla were previously detected in fresh and chilled pork (Yang et al., 2017), traditional fermented fish (Osimani et al., 2019), and dry aged beef (Ryu et al., 2018). There were 91 genera tested in the bacon tissues, including nine dominant genera and 82 non-dominant genera. Among them, five dominant genera (*Aspergillus*, *Candida*, *Debaryomyces*, *Malassezia*, and *Penicillium*) were shared by all bacon tissues. *Aspergillus* was also detected with higher abundance in fresh and chilled pork (Yang et al., 2017). *Debaryomyces* and *Candida* were also present in the traditional Icelandic fermented fish (Osimani et al., 2019), dry aged beef (Ryu et al., 2018), traditional llama meat sausages (Mendoza et al., 2014), and Norwegian dry-cured meat products (Asefa et al., 2009b), and Portuguese cacholeira blood sausage (Belleggia et al., 2020). *Penicillium* was also present in Spanish fermented meat sausage (Lopez-Dıaz et al., 2001), dry aged beef (Ryu et al., 2018), and dry-cured teruel ham (Cavus et al., 2018).

Previous studies have shown that climate, geography, and processing technology had affected the microbial communities in the meat products (Doulgeraki et al., 2012; Yi et al., 2016; Yang et al., 2017; Wang et al., 2018). Because bacon is naturally processed without the use of starter cultures, these fungi may emerge from raw materials or the processing environment. Doulgeraki et al. (2012) have found that raw meat was the main source of microorganisms in bacon preparation. In the process of producing bacon, cured meat is generally hung, smoked and ripened under exposed conditions for a few months; therefore, a variety of endogenous and exogenous microorganisms may be involved. In this study, the climate of Dazhou belongs to the sub-tropical moist monsoon type, which is suitable for the growth of molds and has a great influence on the microbial population in the bacon. Similar results were found in the process of manufacturing dry fermented sausage and other meat products (Scaramuzza et al., 2015; Bernardi et al., 2019; Parussolo et al., 2019). In the present study, *Acremonium*, *Candida*, *Fusicolla*, *Penicillium*, *Tausonia*, and *Trichoderma* were detected in all the bacon tissues, which were previous found in decayed wood (Kim et al., 2006; Guo et al., 2012; Rahmawati et al., 2020). During the processing of Sichuan pork bacon, local producers often use some old wooden utensils, such as chopping board. In addition, bacon is generally ripened in an old house, where the doors, windows, and beams are made of wood. Hence, microorganisms associated with decayed wood in the environment could be involved.

### Effect of Physicochemical Properties of Bacon Tissue on Fungal Communities

**FIGURE 8:**
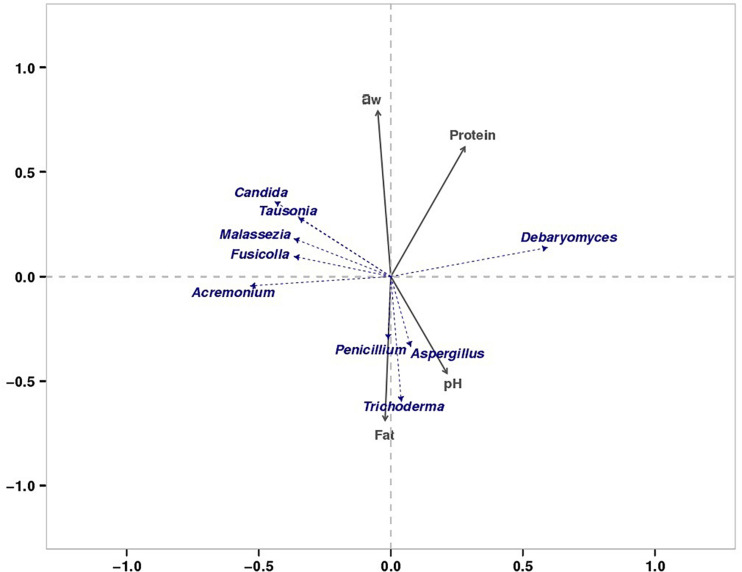
RDA of dominant fungi and major physiochemical properties.

We found that the microorganisms in different parts of bacon exhibited a large degree of biodiversity. LEfSe analysis showed 31 significantly different taxa among the tissues: two phyla, two classes, seven orders, eleven families, and nine genera (Figures 3A,B). The relative abundance of two dominant phyla (Ascomycota and Basidiomycota) significantly differed among the three groups as also did the abundances of nine dominant genera (Table 4). In the present study, the production environment and processing technology of the bacon collected were roughly the same. However, pork meat used in bacon production consists of skin, fat, and lean meat. There are significant differences in the nutritional composition and tissue structure of these tissues (Henry et al., 1963), which may affect the attached microorganisms. As we all known, the growth of microorganisms are greatly affected by the nutritional and environmental factors (Madigan et al., 2011). During the processing and ripening of bacon, different pork tissues serve as selective medium for the growth and reproduction of microorganisms. Different organisms have different preferences for different nutrients and environmental conditions. Therefore, different kinds of microorganisms will be enriched on the surface of different parts of bacon. Similar results were found in the previous study (Greer et al., 1995). Redundancy analysis showed that three dominant fungal genera in bacon (*Aspergillus*, *Penicillium*, and *Trichoderma*) were positively correlated with fat content and pH (Figure 8). *Debaryomyces* showed positive correlation to protein content and pH (Figure 8). *Candida*, *Fusicolla*, *Malassezia*, and *Tausonia* were positively correlated with a_w_, indicating that their growth was profoundly influenced by salt content and moisture content. Previous studies showed that *Penicillium*, *Trichoderma*, and *Aspergillus* were the most prevalent fungi on fresh meat samples and can produce lipase (Abdel-Sater et al., 2017). *Debaryomyces hansenii*, can produce protease which is possible to use in meat production (Tomás et al., 2008). The growth characteristics of these fungi in this study are not yet clear. Therefore, future studies should isolate and grow these fungi to investigate their growth characteristics.

### Characteristics and Interaction of Fungi in Meat Products

Various microbes inhabit traditional foods, and do not exist in isolation (Faust and Raes, 2012; Zhu et al., 2018). The effect of mutual interaction between the co-inhabiting microbes in food ecology is known to play a key part in quality and flavor of food products. Debaryomyces and *Candida* are the most frequently isolated yeasts from meat products (Fleet, 2011). The best-known species of this genus, *Debaryomyces* hansenii showed higher lipolytic activity levels and can contribute to flavor and texture development of meat products (Mendoza et al., 2014). This species can grow under high salt concentrations and is considered an extremophilic organism (Haque et al., 2015). In other reports, *Debaryomyces hansenii* can reduced Ochratoxin A content in meat portions significantly when co-inoculated with *Penicillium nordicum* (Simoncini et al., 2014). *Candida*, a member of the class Saccharomycetes, is a heterogeneous genus that comprises pathogenic species. However, some species of *Candida* play important roles in enhancing the taste and quality of meat products (Martín et al., 2006; Simoncini et al., 2007). *Candida zeylanoides* can inhibit the growth of *Penicillium nordicum* in dry-cured ham and dry fermented sausage (Meftah et al., 2020). *Aspergillus*, the most common genus of mold found in the environment, includes approximately 185 species (Moy et al., 2012). Some species of *Aspergillus*, such as *Aspergillus ruber*, *Aspergillus niveoglaucum*, and *Aspergillus repens*, have a higher proteolytic and lipolytic activity and contribute to the development of the characteristic flavor of the meat product (Alapont et al., 2015). However, some other species of this genus are able to synthesize mycotoxins in a meat substrate when grown under favorable conditions. For example, *Aspergillus flavus* and *Aspergillus parasiticus* commonly found in the meat products can produce aflatoxin B1 (Martin et al., 2004; Cavus et al., 2018). *Penicillium*, a member of the order Eurotiales, is comparable to *Aspergillus* (Visagie et al., 2014). Most species of *Pencillium* fungi are opportunistic and can cause fungal spoilage in fruits and vegetables (Tournas, 2005). Some species of *Penicillium* can produce fungal toxins under certain conditions (Eriksen et al., 2012). However, *Penicillium* is not merely a harmful fungus (Perrone and Susca, 2017). Numerous species of this genus produce extracellular enzymes such as glucoamylase, lipase, pectinase, and alpha-amylase, which may affect product quality (Chellegatti et al., 2000; Balkan and Ertan, 2005; Bancerz et al., 2006; Magistà et al., 2017). *Penicillium roquefortii* and *Penicillium camambertii* are two common species used to produce cheese (Toelstede and Hofmann, 2009; Fairclough et al., 2011). *Penicillium chrysogenum* can produce glucose oxidase, which can help to prevent fungal decay and be used as a preservative (Konishi et al., 2013; Garrigues et al., 2018). *Malassezia*, a yeast-like fungus, which lack the ability to synthesize medium-chain and long-chain fatty acids (Roberta et al., 2020). It is generally found on normal skin, which causes opportunistic infections. Some species of *Malassezia* were also detected in some refrigerators (Ye et al., 2019). In the present study, the exact function of these fungi was not determined. Therefore, future studies should isolate these fungi and investigate their interactions by co-culture or other methods.

## Conclusion

In this study, five dominant genera (*Aspergillus, Candida, Debaryomyces, Malassezia*, and *Penicillium*) were shared by all bacon tissues, and a total of 31 operational taxonomic units at different levels significantly differed between different tissues. The results also showed that fungal communities in different bacon tissue were significantly different. Results of redundancy analysis and network analysis indicated that physicochemical properties of bacon tissue were a crucial factor influencing the fungal diversity and communities of homemade traditional Sichuan bacon. This study will improve our understanding of the fungal diversity and communities in traditional pork bacon. Further investigations are required to identify these isolate from the product and to reveal the interaction between spoilage and microbes.

## Data Availability Statement

The datasets presented in this study can be found in online repositories. The names of the repository/repositories and accession number(s) can be found below: https://www.ncbi.nlm.nih.gov/, PRJNA610642.

## Author Contributions

HQ is co-first author. MZ, HQ, and WZ contributed conception and design of the study. ZZ, PW, and YZ performed the statistical analysis. MZ and HQ wrote the first draft of the manuscript. All authors contributed to manuscript revision, read, and approved the submitted version.

## Conflict of Interest

The authors declare that the research was conducted in the absence of any commercial or financial relationships that could be construed as a potential conflict of interest.

## Footnotes

1 http://qiime.org/
